# Case Report: Metreleptin and SGLT2 Inhibitor Combination Therapy Is Effective for Acquired Incomplete Lipodystrophy

**DOI:** 10.3389/fendo.2021.690996

**Published:** 2021-05-31

**Authors:** Ayako Nagayama, Kenji Ashida, Miki Watanabe, Kanoko Moritaka, Aya Sonezaki, Yoichiro Kitajima, Hirokazu Takahashi, Satoko Yoshinobu, Shimpei Iwata, Junichi Yasuda, Nao Hasuzawa, Shuichi Ozono, Seiichi Motomura, Masatoshi Nomura

**Affiliations:** ^1^ Division of Endocrinology and Metabolism, Department of Internal Medicine, Kurume University School of Medicine, Kurume, Japan; ^2^ Eguchi Hospital, Ogi, Japan; ^3^ Division of Hepatology, Diabetes Mellitus, and Endocrinology, Department of Internal Medicine, Saga University, Saga, Japan; ^4^ Department of Pediatrics and Child Health, Kurume University School of Medicine, Kurume, Japan

**Keywords:** lipodystrophy, SGLT2 inhibitor, metreleptin, hypertriglyceridemia, diabetes mellitus

## Abstract

Childhood cancer survivors (CCSs) who have undergone bone marrow transplantation with systemic chemotherapy and whole-body irradiation often experience impaired glucose tolerance with marked insulin resistance. Incomplete acquired diabetic lipodystrophy should be considered as a late complication of bone marrow transplantation. A 24-year-old Japanese female patient with incomplete acquired lipodystrophy, a CCS of acute lymphocytic leukemia at the age of 3 years, was treated for diabetes mellitus and dyslipidemia at our hospital. Administration of multiple daily insulin injections (70 units/day), and oral administration of 500 mg/day metformin, 15 mg/day pioglitazone, and 200 mg/day bezafibrate had proven ineffective for her metabolic disorders. Subcutaneous administration of metreleptin improved her insulin resistance and hypertriglyceridemia within a month; however, it failed to maintain adequate plasma glucose levels in the long term. When oral administration of 10 mg/day empagliflozin was added to the metreleptin supplementation, her HbA1c value (National Glycohemoglobin Standardization Program) improved from 11% to 8%, which was maintained for an additional 18 months. This is the first case report of incomplete lipodystrophy that shows efficacy of a combination therapy with metreleptin and a sodium glucose cotransporter 2 (SGLT2) inhibitor for the treatment of diabetes and dyslipidemia. An SGLT2 inhibitor attenuates hyperglycemia through urinary glucose excretion and has been suggested to enhance lipid catabolism in the extra-adipose tissues, especially in the liver and skeletal muscles. Furthermore, metreleptin supplementation could enhance the action of the SGLT2 inhibitor by promoting satiety and lipolysis through the central nervous system. Combination therapy with metreleptin and an SGLT2 inhibitor was suggested to recover the volume of adipose tissue, possibly through improvement of insulin resistance in the adipose tissue. This report highlights the pathophysiological mechanism of an SGLT2 inhibitor in the improvement of glucose metabolism in non-healthy lean CCSs with insulin resistance. Administration of SGLT2 inhibitor, along with metreleptin supplementation, could be a good alternative therapy for diabetic lipodystrophy observed in CCSs.

## Introduction

Childhood cancer survivors (CCS) are at a high risk of cardiovascular events and mortality (1, 2), even after they have overcome their critical malignancies. CCSs often experience impaired glucose tolerance with marked insulin resistance as a late complication of hematopoietic stem cell transplantation and whole-body irradiation during childhood (3). Acquired incomplete lipodystrophy is considered as the pathogenesis for these conditions (4). Accordingly, supplementation with recombinant leptin, metreleptin, is a reasonable therapy for CCS for the improvement of their lipid and blood glucose profiles (5–7), and for the prevention of critical cardiovascular complications.

Disturbance in both white and brown adipose tissues has been suggested to occur during radiation therapy-related acquired incomplete lipodystrophy (4), although white adipose tissue abnormalities are the predominant disorder in congenital generalized lipodystrophy (8). Administration of a sodium glucose cotransporter 2 (SGLT2) inhibitor has been suggested as a candidate therapy for glucose impairment in a patient with congenital generalized lipodystrophy (9). SGLT2 inhibitor administration reduces hyperglycemia and fat accumulation in adipose tissues by increasing urinary glucose excretion in patients with type 2 diabetes mellitus (T2DM) (10). Additionally, an SGLT2 inhibitor has been suggested to improve glucose impairment through changes in glucose and lipid metabolisms in both adipose and extra-adipose tissues (11, 12). However, the precise mechanism of the beneficial effects of the SGLT2 inhibitor on lipodystrophy remains to be clarified.

We present a case of acquired incomplete diabetic lipodystrophy wherein the patient was treated with a combination of metreleptin supplementation and an SGLT2 inhibitor. We have previously reported the effect of metreleptin administration on lipid and glucose metabolisms in this case (5). An additional 2-year observation revealed dysregulation of hyperglycemia and continuous amelioration of hyperlipidemia. Herein, we observed that SGLT2 inhibitor administration in addition to metreleptin supplementation could regulate the patient’s hyperglycemia and hyperlipidemia. An improvement was observed in her glucose and lipid profiles for an additional 18 months. Furthermore, recovery of adipose accumulation in the subcutaneous and visceral adipose tissues was demonstrated. SGLT2 inhibitor administration has the potential to be a suitable alternative for the treatment of major abnormalities in glucose and lipid metabolism and body composition in CCSs.

## Case Description

**Figure 1:**
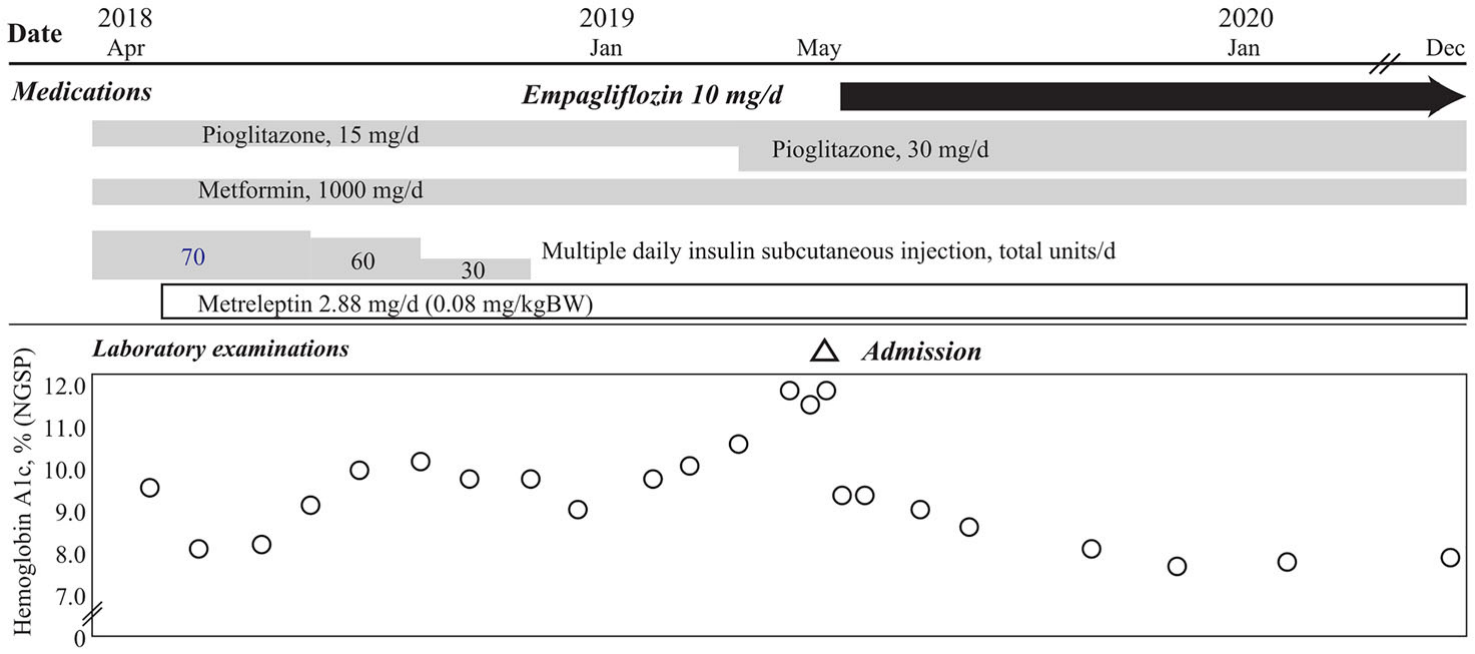
Clinical course of the patient in this case. Hemoglobin A1c levels of the patient in this case show rapid reduction after oral administration of empagliflozin, with continuation of 15 mg/day pioglitazone and 1000 mg/day metformin, and 2.88 mg/day of subcutaneous metreleptin supplementation, with termination of insulin injection. The HbA1c level is maintained at ~8.0%, 18 months after empagliflozin initiation.

A 24-year-old Japanese female patient presented to our endocrinology center with hyperglycemia, and a 13-year history of acquired incomplete diabetic lipodystrophy with hypertriglyceridemia and non-alcoholic fatty liver disease (5). Systemic chemotherapy and whole-body irradiation before allogeneic stem cell transplantation for acute lymphocytic leukemia at 3 years of age were considered as possible reasons for lipodystrophy development (3, 5). Administration of 500 mg/day metformin and 15 mg/day pioglitazone, along with multiple daily subcutaneous injections of high-dose insulin (70 units/day) had failed to improve her hyperglycemia (**Figure 1**). Moreover, oral administration of 200 mg/day bezafibrate had failed to improve the hypertriglyceridemia. However, subcutaneous administration of metreleptin (0.08 mg/kg bodyweight/day) markedly improved her hypertriglyceridemia from 3897 mg/dL to 1828 mg/dL (reference range, 30–149 mg/dL) and the insulin resistance; the glucose infusion rate (GIR) during euglycemic glucose clamp examination (<5.7 mg/kg/min indicates insulin resistance) increased from 2.1 mg/kg bodyweight/min to 3.2 mg/kg bodyweight/min in 1 month. Her glycemic control improved, and the HbA1c value attenuated rapidly from 9.6% to 8.1% (National Glycohemoglobin Standardization Program: NGSP) (reference range, 4.6–6.2%) (5); however, no further consistent improvement was observed, and her HbA1c levels increased to 11.3% before empagliflozin initiation (**Figure 1** and **Table 1**).

**Table 1:** Laboratory findings of glucose metabolisms evaluated pre- and post-treatment with an SGLT2 inhibitor.

Variables, unit	Value		Reference range	
	Pre-treatment	Post-treatment		
**Diabetes mellitus profile**			
Plasma glucose, mg/dL	215	98	73–109
HbA1c, % (NGSP)	11.3	7.8	4.9–6.0
Serum C-peptide, ng/dL	3.9	3.3	0.8–2.5
3-OHBA, μmol/L	66	90	0–74.0
HOMA-IR	6.37	2.46	
Glucagon loading test^†^			
ΔC-peptide, ng/dL	2.92	2.10	
Urinalysis			
Albumin, mg/day	1963.5	1051.5	
Glucose, g/day	29.14	24.64	

^†^ΔC-peptide is defined as an elevated value of the serum C-peptide level measured 6 minutes after the administration of 1-mg glucagon intravenous injection when compared with the baseline level.

HbA1c, hemoglobin A1c; 3-OHBA, 3-hydroxybutyric acid; GIR, glucose infusion rate; NGSP, National Glycohemoglobin Standardization Program.

**Figure 2:**
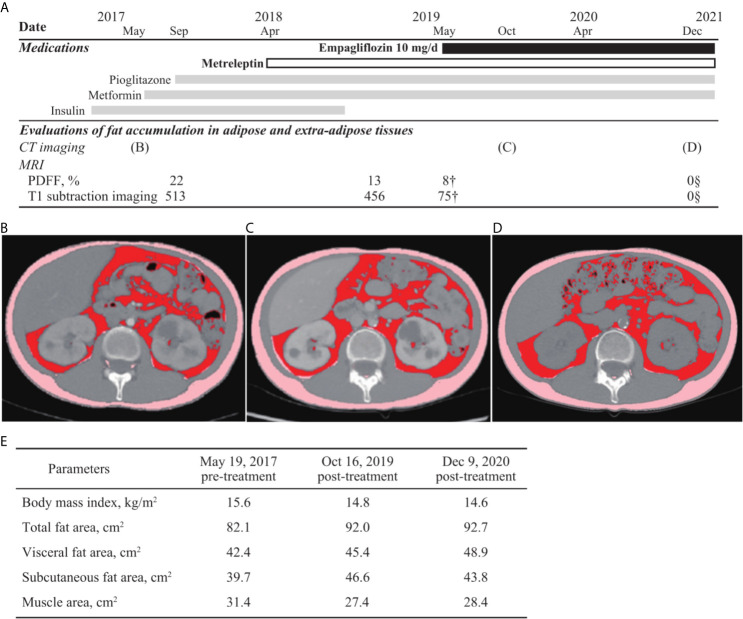
Evaluations of fat accumulation in the adipose and extra-adipose tissues. Body fat accumulation has been evaluated during the clinical course. **(A)** The time of evaluation using computed tomography (CT) imaging and magnetic resonance imaging as well as clinical time course of medications are presented. Fat accumulation in liver is decreased and eventually, not detected using the proton density fat fraction (PDFF) method and T1 subtraction imaging. Areas of visceral, subcutaneous, and total fat are increased, observed in the CT imaging analysis between before **(B)** and after **(C, D)** metreleptin and empagliflozin administrations, although muscle areas are not increased **(E)**. Additionally, changes in fat volume and body mass index between before- and after-combination therapy using SGLT2 inhibitor and metreleptin administration are demonstrated. CT, computed tomography; PDFF, proton density fat fraction.

After 1 year of metreleptin administration, oral administration of 10 mg/day empagliflozin was initiated, as her HbA1c level was still high at 11% (NGSP). Her glycemic control improved immediately, and the HbA1c level decreased to ~8% (NGSP). On physical examination, her bodyweight and body mass index of 30.5 kg and 15.6 kg/m^2^, respectively, before administration of metreleptin and empagliflozin, had reduced to 28.6 kg and 14.6 kg/m^2^, respectively, following 18 months from empagliflozin initiation and continuous metreleptin supplementation. The area under the curve and fluctuations in the daily plasma glucose, measured using a flash glucose monitoring (FGM) system, demonstrated significant improvements (**Supplementary Figure 1**). Notably, body composition analysis using computed tomography imaging disclosed an increase in visceral and subcutaneous fat accumulation, with reduced liver steatosis and unchanged muscle areas (**Figure 2**). Additionally, her insulin resistance and liver steatosis, evaluated using proton density fat fraction and T1 subtraction imaging on magnetic resonance imaging, improved (13) (**Figure 2**). Furthermore, her triglyceride level had decreased to 652 mg/dL (**Table 2**). For an additional 18 months from SGLT2 inhibitor initiation, her HbA1c level remained steady at 7.8% (NGSP); no specific adverse events, including urinary tract infection, were observed (**Figure 1** and **Table 1**). Evaluations using a questionnaire that comprised a visual analogue scale analysis revealed enhanced satiety in the patient; her appetite had reduced to 70% of what it was prior to metreleptin administration. Based on the ideal body mass index for the patient and her routine activities, a diet therapy with an intake of 1380 kcal/day was recommended (14). Administration of the SGLT2 inhibitor did not alter her satiety or adherence to the therapies. Her adherence to the combination therapy was well-preserved and her vigor had increased.

**Table 2:** Results of laboratory examinations pre- and post-treatment with an SGLT2-inhibitor.

Variables, unit	Value		Reference range
	Pre-treatment	Post-treatment	
**Serum chemistry**			
Albumin, g/dL	4.0	4.3	4.1–5.1
AST, IU/L	17	16	13–30
ALT, IU/L	21	11	7–30
γ-Glutamyl transpeptidase	26	19	9–32
**Lipid profile**			
LDL cholesterol, mg/dL	80	136	65.0–139.0
HDL cholesterol, mg/dL	29	32	40–103
RLP cholesterol, mg/dL	88.7	31.5	≤ 7.5
Triglyceride, mg/dL	1816	652	30–149
Apolipoprotein			
A-I, mg/dL	102	110	122–161
A-II, mg/dL	35.4	33.3	24.6–33.3
B, mg/dL	159	145	69–105
C-II, mg/dL	31.4	16.1	1.5–3.8
C-III, mg/dL	85.3	40.2	5.4–9.0
E, mg/dL	23.3	8.6	> 36
Leptin, ng/mL	10†	856‡, 240	2.5–21.8
Adiponectin, μg/mL	0.3†	1.79	> 4.0

Blood surrogate markers related to fatty liver or lipid profile are listed. The values described for before and after SGLT2 inhibitor administration are presented as pre-treatment and post-treatment, respectively. Both pre-treatment and post-treatment values are those measured with the administration of metreleptin, except for the measurement of leptin and adiponectin. ^†^Measured before metreleptin administration. ^‡^Measured after metreleptin administration and before SGLT2 inhibitor administration.

## Discussion

We observed the first case of acquired diabetic lipodystrophy in which hyperglycemia improved with administration of SGLT2 inhibitor in addition to subcutaneous metreleptin supplementation. Additionally, combination therapy with metreleptin and empagliflozin decreased adipose accumulation in the extra-adipose tissues, and increased it in the adipose tissues. This study revealed the potential benefits of using SGLT2 inhibitors for non-obese patients with diabetes, especially in CCSs with lipodystrophy.

Combination therapy with SGLT2 inhibitor and metreleptin supplementation has the potential to improve glycemic control in patients with acquired diabetic lipodystrophy. The role of ectopic adipose accumulation in insulin resistance has been indicated in both complete and incomplete lipodystrophies, which is consistent with the findings of previous literature (9). Administration of an SGLT2 inhibitor increased urinary glucose excretion, and significantly improved the daily plasma glucose fluctuation and fasting plasma glucose levels (**Table 1**, **Figure 1**, and **Supplementary Figure 1**) (12, 15). Administration of an SGLT2 inhibitor promotes glucagon secretion. Chronic promotion of glucagon secretion activates mitochondrial oxidative phosphorylation, which leads to the attenuation of liver steatosis and insulin resistance (16). Moreover, a previous study has described a case of complete diabetic lipodystrophy wherein hyperglycemia rapidly improved after SGLT2 inhibitor administration (9). The glucose metabolism impairment in our case improved to approximately the same level as that in the complete lipodystrophy case; the HbA1c level of our patient changed from 11.3% to 7.6% in 7 months, and in the previously reported case (9), from 9.3% to 6.9% in 6 months. We confirmed consistent improvement in the glucose metabolism with regard to insulin resistance and plasma glucose variability, evaluated by GIR and the homeostasis model assessment of insulin resistance (HOMA-IR), and FGM, respectively. Although metreleptin supplementation failed to improve the hyperglycemia in this study before SGLT2 inhibitor administration, a previous study demonstrated consistent improvement in glucose levels in a patient with CCS-related diabetic dystrophy (7). Leptin administration has been shown to improve insulin sensitivity through the activation of adenosine monophosphate-activated protein kinase (17). Furthermore, metreleptin supplementation enhances satiety through the hypothalamus (18), while SGLT2 inhibitor administration enhances appetite as an adverse effect (15). Considering these findings, sufficient leptin activity is required in patients with lipodystrophy for effective diabetes mellitus therapy using an SGLT2 inhibitor.

SGLT2 inhibitor administration under sufficient leptin function was suggested to decrease adipose accumulation in the extra-adipose tissues, and increased it in the adipose tissues. Combination therapy with SGLT2 inhibitor and metreleptin increased both subcutaneous and visceral adipose volume in our patient, who had low adiposity. Administration of an SGLT2 inhibitor may demonstrate adipose redistribution through attenuation of insulin resistance and lipolysis in the extra-adipose tissues (11). Additionally, a recent study has described a case of CCS-related acquired partial lipodystrophy with high adiposity wherein adiposity in the fat tissue was decreased after metreleptin administration. Furthermore, metreleptin supplementation activates brown adipose tissue and lipolysis in some regions, including extra-adipose tissues, through sympathetic nerve stimulation (18, 19). In this context, the combination therapy should lead to the redistribution of adipose tissues in the patients with CCS-related lipodystrophy. Notably, disturbance in both white and brown adipose tissues has been observed in CCSs with lipodystrophy, although white adipose tissue insufficiency is observed predominantly in patients with inherited complete lipodystrophy (8). Furthermore, both visceral and subcutaneous fat volumes were recovered using the combination therapy of metreleptin and SGLT2 inhibitor administration in this case. SGLT2 inhibitor with metreleptin supplementation may improve glucose metabolism impairment, compensating for the brown and white adipose tissue dysfunction and preserving skeletal muscle volume (9–11) in CCSs with diabetic lipodystrophy.

SGLT2 inhibitor could improve the prognosis of CCSs through risk reduction of their cardiovascular events. The patient in this case had multiple risk factors for cardiovascular disease, including diabetes mellitus with insulin resistance and high daily glucose fluctuation, hypertriglyceridemia, non-alcoholic fatty liver disease, and low adiponectin level. Empagliflozin has been shown to improve cardiovascular outcomes such as the all-cause mortality rate of patients with T2DM (20). Furthermore, several advantages of SGLT2 inhibitor use have been reported, which includes reduction in hyperinsulinemia in patients with T2DM (21). Additionally, improvements in daily glucose fluctuation, which promote cardiovascular events (22), have been studied (11, 23). Both amelioration of insulin resistance and daily glucose fluctuation are plausible pathophysiological mechanisms of reducing the risk of a cardiovascular event. Ectopic adiposities, especially as liver and muscle steatosis, can be the targets of SGLT2 inhibitor use in non-obese patients with T2DM (11, 24). CCSs, with accumulating re-distributed ectopic fat from the adipose tissues to the liver and muscles, are good candidates to explain the pathophysiological mechanism of SGLT2 inhibitors, beyond its glucose-lowering effect.

Our case study had few limitations. Administration of the SGLT2 inhibitor alone might have improved the glucose impairment and dyslipidemia in this case. Additional observation is required to demonstrate the long-term effectiveness and safety of SGLT2 inhibitors in CCSs. We believe that further case studies or large-scale studies might confirm our conclusion.

## Conclusion

Hyperglycemia associated with acquired diabetic lipodystrophy improved with the administration of an SGLT2 inhibitor in addition to metreleptin supplementation. This study revealed the potential benefits of combination therapy using metreleptin and an SGLT2 inhibitor for non-obese patients with diabetes, especially CCSs with lipodystrophy. SGLT2 inhibitors could improve the prognosis of CCSs through risk reduction of their cardiovascular events. Further, this study revealed the pathophysiological mechanisms of glucose metabolism in CCSs and non-healthy non-obese patients with insulin resistance.

## Data Availability Statement

The original contributions presented in the study are included in the article/**Supplementary Material**. Further inquiries can be directed to the corresponding author.

## Ethics Statement

The studies involving human participants were reviewed and approved by Kurume University Hospital. The patients/participants provided their written informed consent to participate in this study. Written informed consent was obtained from the individual(s) for the publication of any potentially identifiable images or data included in this article.

## Author Contributions

AN collected the data and wrote the first draft of the manuscript with the support of KA and MN. KA, YK, HT, and MN reviewed the manuscript. AN, KA, MW, KM, AS, YK, HT, SY, SI, JY, NH, SO, SM, and MN discussed the case and approved the final manuscript. All authors contributed to the article and approved the submitted version.

## Conflict of Interest

The authors declare that the research was conducted in the absence of any commercial or financial relationships that could be construed as a potential conflict of interest.
