# Supplementary material for: Case Report: Metreleptin and SGLT2 Inhibitor Combination Therapy Is Effective for Acquired Incomplete Lipodystrophy
Source: Front Endocrinol (Lausanne). 2021 May 31;12:690996. doi: 10.3389/fendo.2021.690996 (PMC8201990; doi:10.3389/fendo.2021.690996)
Supplement: Supplementary file 1 [file DataSheet_1.docx]

Supplementary Material


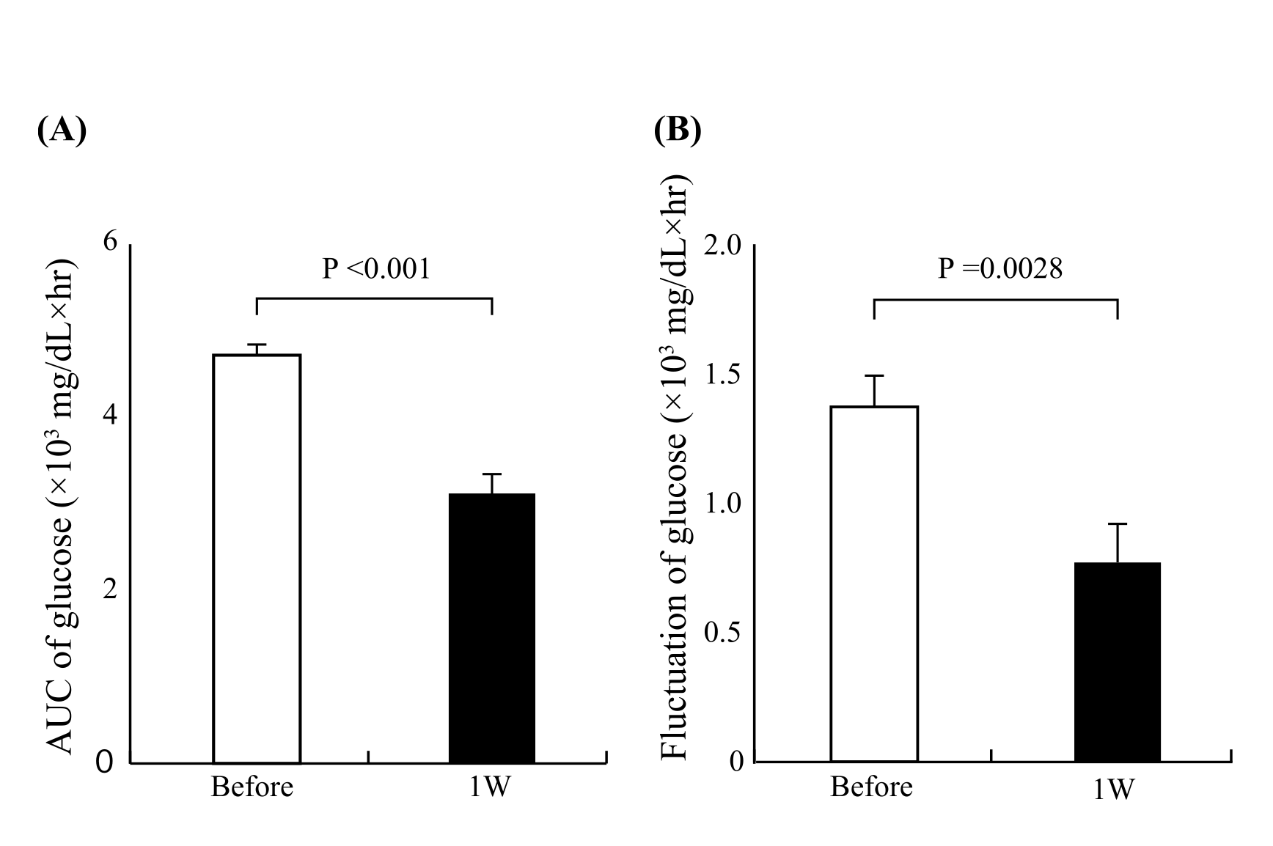


**Supplementary Figure 1.** **Attenuations of the area under the curve (AUC) and fluctuations in daily plasma glucose (PG) levels after empagliflozin administration**

(A) The AUC of PG is significantly reduced by the 1-week administration of empagliflozin. The values are presented as the mean of sequential 3-day ± standard deviation before (4715±127 mg/dL×hr) and after 1-week administration of empagliflozin (3119±238 mg/dL×hr). (B) Fluctuations in daily PG levels are significantly reduced after empagliflozin administration. Fluctuations in glucose levels: summation area of difference between the mean and the actual glucose levels in daily glucose profiles are presented as the mean of sequential 3-day ± standard deviation before (1366 ± 122 mg/dL×hr) and after 1-week administration of empagliflozin (767 ± 148 mg/dL×hr). Abbreviations: AUC, area under the curve; PG, plasma glucose.
